# Chemotherapy of metastatic triple negative breast cancer: Experience of using platinum-based chemotherapy

**DOI:** 10.18632/oncotarget.5654

**Published:** 2015-10-03

**Authors:** Jian Zhang, Minhao Fan, Jie Xie, Zhonghua Wang, Biyun Wang, Sheng Zhang, Leiping Wang, Jun Cao, Zhonghua Tao, Ting Li, Xichun Hu

**Affiliations:** ^1^ Department of Medical Oncology, Fudan University Shanghai Cancer Center, Department of Oncology, Shanghai Medical College, Fudan University, Shanghai 200032, China

**Keywords:** platinums, metastatic breast cancer, triple-negative, chemotherapy

## Abstract

The results of recent studies investigating the role of platinum-based chemotherapy (PBCT) in metastatic triple-negative breast cancer (mTNBC) were conflicting. We retrospectively investigated a large cohort (*n* = 379) of mTNBC to re-evaluate the role of platinums. Longer PFS was found in patients with PBCT than those with non-PBCT (7.8 vs. 4.9 months, *P* < 0.001) as first-line chemotherapy, but no statistical difference of OS was observed. Compared with other kinds of platinum, cisplatin-based regimens as the first-line chemotherapy showed better PFS (8.0 vs. 4.3 months, *P* = 0.03) and better ORR. Introduction of ≥2 lines, rather than 1 line, of PBCT can result in better OS when compared with no introduction of PBCT during the whole treatment. If considering the timing of intervention of PBCT, first-line introduction and later line introduction of PBCT did not make any difference in OS among patients with only one line PBCT during the whole treatment. We concluded that PBCT with only 1 line during the whole treatment might not be necessary for unselected mTNBC with the exception of an urgent demand to control disease or symptoms, however, ≥2 lines of PBCT did prolong OS.

## INTRODUCTION

Triple-negative breast cancer (TNBC) which accounts for ∼12–20% of all breast cancers is a clinical challenge as this subtype is associated with an increased rate of recurrence, shorter disease-free intervals (DFI), earlier visceral metastasis, and poorer survival compared with other breast cancer subtypes [1, 2]. The median survival for metastatic TNBC (mTNBC) ranges from 6 to 13.3 months [3–6]. Cytotoxic chemotherapy is the mainstay treatment for mTNBC and no dedicated biological agents are available. Targeted therapies including DNA repair agents, PARP or EGFR inhibitors, anti-angiogenic agents, or checkpoint kinase 1 (Chk1) inhibitors (with or without chemotherapy) have not substantially improved mTNBC outcomes [7, 8].

Gene expression analysis demonstrates that the molecular signature of TNBC generally overlaps with basal-like breast cancer (BLBC), with concordance rates of 70–90% [9–12], TNBC is also associated with BRCA-related breast cancer. The incidence of BRCA mutations in TNBC varies from 16–42% and IHC-based studies classify 80–90% of BRCA1-associated tumors as TNBC and/or BLBC [13, 14]. Due to these similarities, it has been hypothesized that the DNA repair defects that sensitize BRCA-mutated and BRCA-like breast cancer tumors to platinum may also be present in TNBC, indicating that platinum-based chemotherapy (PBCT) may be an effective treatment option for this subset of breast cancer [15].

During the past few years, several studies have been conducted to study the role of PBCT in the mTNBC setting, but results were conflicting. Some data support the use of platinum (mainly cisplatin-based) [16–18]. While some are discouraging, for example, the TNT study compared carboplatin to docetaxel therapy in unselectted metastatic or recurrent locally advanced TNBC or BRCA1/2 positive breast cancer but similar efficacy was observed in terms of overall response rate (ORR [31.4% vs. 35.6%, *p* = 0.44]), PFS (3.1 vs. 4.5 months, *p* = 0.29) and overall survival (OS [12.4 vs. 12.3 months, *p* = 0.31]) [19]. Even with a first-line gemcitabine and carboplatin combination regimen, PFS was only 4.6 months, which did not appear significantly superior to historical data [6, 20]. Likely, cisplatin is more efficacious than carboplatin [21, 22]. We need more evidence in unselected mTNBC patients to confirm that PBCT is more efficacious than non-PBCT, and whether these differences are due to types of platinums, and whether later-line treatment with platinums influence OS. We then retrospectively reviewed a large cohort of mTNBC patients at Fudan University Shanghai Cancer Center (FUSCC) to re-evaluate of the role of platinums and compared potential prognostic factors for first-line ORR and PFS and OS.

## RESULTS

### Patient characteristics

Between July 2000 and March 2014, patients having received at least one line of chemotherapy in the metastatic setting, including 309 of 379 patients (81.5%) who received one or more platinum-based regimens and 70 patients (18.5%) who received non-platinum regimens, were identified from our database. Table 1 depicts their characteristics and treatments. Therapies administrated were summarized in Table 2.

**Table 1:** Patient characteristics at time of metastases diagnosis (*n* = 379)

Characteristics	No.	%
Median age (years, range)	49 (25–76)	
<40	73	19.3
≥40	306	80.7
Pathological types		
Invasive ductal carcinoma	355	93.7
Others	24	6.3
Menstruation status		
Pre- or peri-menopausal	179	47.2
Post-menopausal	200	52.8
ECOG performance status		
0	87	23.0
1	281	74.1
≥2	11	2.9
Time between breast surgery and recurrent disease(months, range)	14.3 (0–217.0)	
<12	133	35.1
≥12	224	59.1
de novo metastatic	22	5.8
Number of metastatic organ sites		
1	126	33.2
2	143	37.7
≥ 3	110	29.0
Metastatic sites		
Lymph node	230	60.7
Lung	163	43.0
Chest wall	108	28.5
Bone	99	26.1
Liver	76	20.1
Pleura	32	8.4
Brain	11	2.9
Breast	4	1.1
Type of metastasis		
Visceral	212	55.9
Non-visceral	167	44.1
Adjuvant or neo-adjuvant chemotherapy		
Yes	341	90.0
Anthracyclines only	106	28.0
Taxanes only	24	6.3
Both Anthracyclines and Taxanes	192	50.7
With Platinum	36	9.5
Without Platinum	305	80.5
No	38	10.0

Abbreviations: ECOG, Eastern Cooperative Oncology Group

**Table 2:** Summary of therapies in the metastatic setting

	No.	%
**Total lines of therapy received (*n* = 379)**		
1	83	21.9
2	111	29.3
3	85	22.4
4	50	13.2
≥5	44	11.6
Uncertain	6	1.6
**Total lines of PBCT received (*n* = 379)**		
Yes	309	81.5
1	195	51.5
2	85	22.4
3	22	5.8
4	7	1.8
No	70	18.5
**Line of the first introduction of PBCT (*n* = 309)**		
1^st^	218	70.6
2^nd^	64	20.7
3^rd^	20	6.5
4^th^	4	1.3
≥5^th^	3	1.0
**Regimen of the first introduction of PBCT (*n* = 309)**		
Gemcitabine + cisplatin	171	55.3
Vinorelbine + cisplatin	30	9.7
Docetaxel + cisplatin	29	9.4
mFOLFOX6	25	8.1
Vinorelbine + oxaliplatin	19	6.1
Abraxane + cisplatin	16	5.2
Carboplatin-based and Others	19	6.1
**Regimen of the second introduction of PBCT (*n* = 114)**		
mFOLFOX6	29	25.4
Vinorelbine + oxaliplatin	28	24.6
Gemcitabine + cisplatin	9	7.9
Paclitaxel + carboplatin	7	6.1
Abraxane + carboplatin	6	5.3
Others	35	30.7

Abbreviations: PBCT, platinum-based chemotherapy

Among the 364 patients with available PFS data, 218 received PBCT as their first line chemotherapy for mTNBC and 146 patients received non-PBCT correspondingly. The median follow-up was 15.9 months (range, 2.0–127.4 months) by the time of data lock (March 20, 2014). As the date of data cut off, 319 events of disease progression among the 364 patients as first line chemotherapy and 267 deaths in total of the 379 had been observed, with 5 patients (1.3%) were lost to follow-up. Ninety percent patients received neo-adjuvant or adjuvant chemotherapy. Prior exposure rates were 78.7% to anthracyclines and 57.0% to taxanes, with 9.5% exposed to platinum in the neo-adjuvant or adjuvant setting. Table 2 summarizes therapies in the metastatic setting with respect to types and strategies for PBCT used. On average, mTNBC patients received 2.7 lines of chemotherapy in the metastatic setting (range, 1–9).

### Response and survival

**Figure 1:**
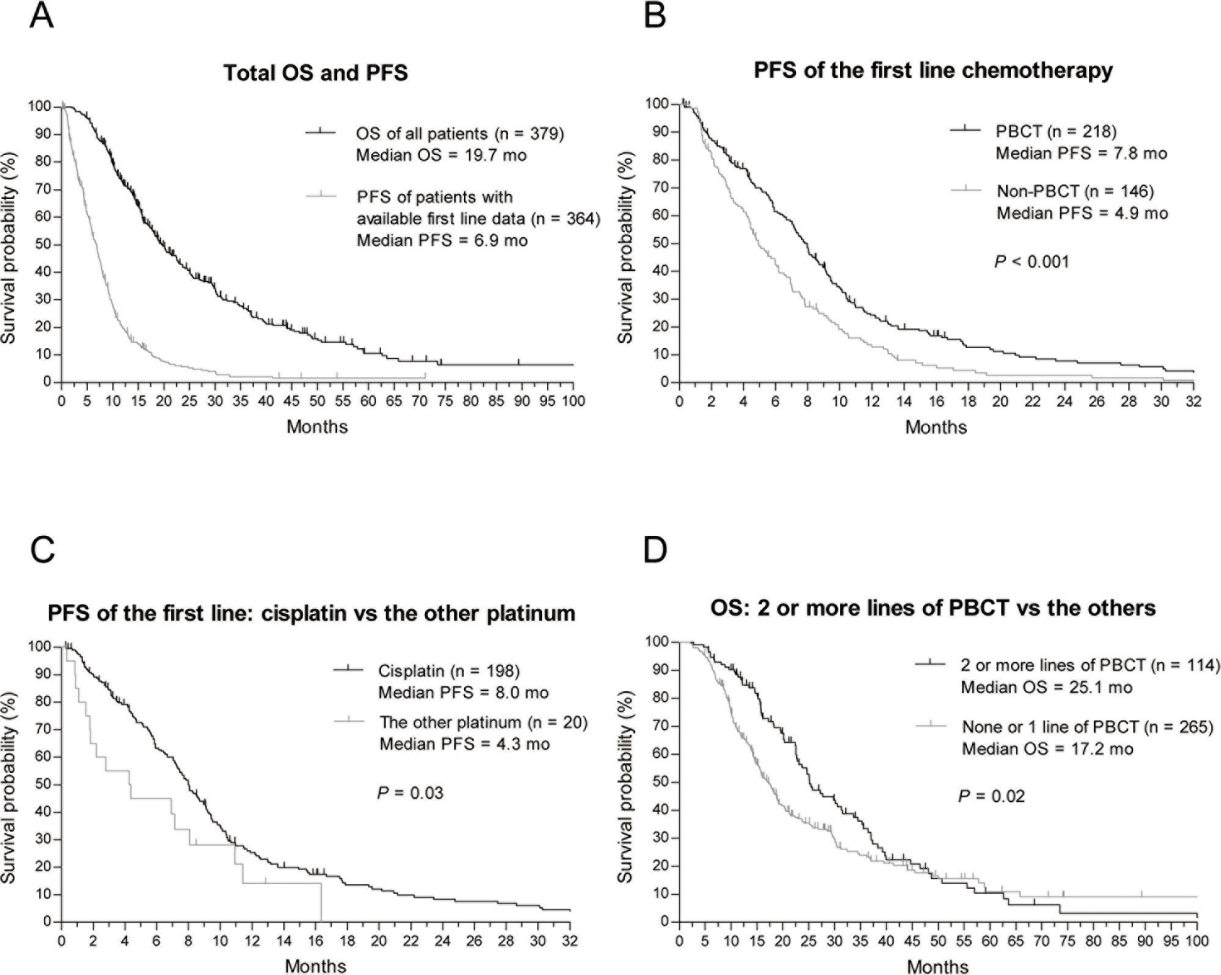
**A.** Kaplan-Meier estimates of OS for the whole cohort and PFS for patients with available first-line data **B.** Kaplan-Meier estimates of PFS for different chemotherapy in the first-line setting (PBCT vs. non-PBCT) **C.** Kaplan-Meier estimates of PFS for different platinums in first-line PBCT (cisplatin vs. other platinums) **D.** Kaplan-Meier estimates of OS for different total lines of PBCT (≥ 2 lines vs. 0–1 line).

By the time of data lock, the median OS of all 379 patients was 19.7 months (95% CI, 17.2 to 22.1). Among the 364 patients with available first-line data, the median PFS was 6.9 months (95% CI, 6.1 to 7.7) (Fig. 1A), with the ORR of 47.5% (26 CR, 147 PR). Longer PFS observed in patients with PBCT compared to those with non-PBCT (median PFS, 7.8 months vs. 4.9 months, *P* < 0.001) as first-line chemotherapy for mTNBC (Fig. 1B). The ORR was also statistically higher in the first-line PBCT group than in the non-PBCT group (57.3% vs. 32.9%, *P* < 0.001). No statistical difference in OS was observed between these two groups (median OS, 19.6 months vs. 19.2 months, *P* = 0.82).

Compared with other forms of platinum, cisplatin-based regimens (cisplatin + taxanes/vinorelbine/gemcitabine) as first-line chemotherapy offered better PFS (median PFS, 8.0 months vs. 4.3 months, *P* = 0.03; Fig. 1C) and better ORR (59.6% vs. 35.0%, *P* = 0.03), but no benefit to OS was observed (median OS, 25.1 months vs. 19.6 months, *P* = 0.69).

A numerical but not a significant difference was observed (median OS, 20.3 months vs. 15.7 months, *P* = 0.87) in terms of OS between patients with an introduction and those with no introduction of PBCT during treatment. However, more lines of intervention with PBCT offered better OS outcomes. For all 379 patients, those who received 2 or more lines of PBCT in their metastatic setting had longer OS than those who received none or only one line of PBCT (median OS, 25.1 months vs. 17.2 months, *P* = 0.02; Fig. 1D). This advantage remained with the 309 patients with PBCT in the metastatic setting, and better OS was seen for those with ≥2 lines of PBCT compared to those with only one line of PBCT (median OS, 25.1 months vs. 17.2 months, *P* = 0.01). Regarding timing of the PBCT intervention, first-line and later line introduction of PBCT was not different with respect to OS (20.2 months vs. 15.6 months, *P* = 0.15) among patients with only one line of PBCT during the treatment.

Other variables significantly influencing PFS in the univariate analysis included ECOG score, time between breast surgery and number of metastatic organ sites. Besides receiving ≥2 lines of PBCT, the univariate analysis also indicated that post-menopausal, time between breast surgery ≥12 months, no visceral metastasis and less number of metastatic organ sites were significantly associated with longer OS. Previous use of platinum as adjuvant or neo-adjuvant did not significantly influence the PFS or ORR of first-line chemotherapy in the metastatic setting (median PFS, 5.1 months vs. 6.9 months, *P* = 0.29; ORR, 47.1% vs. 47.6%, *P* = 0.95).

### Factors independently predicting treatment outcome

Using a binary logistic regression model, only first-line PBCT (HR, 0.36, 95% CI 0.21 – 0.61; *P* < 0.001) was identified as independent factors for predicting first-line ORR.

Cox proportional hazards model was used to determine the hazard ratios of the above-mentioned variables for predicting the risk of disease progression or death for mTNBC patients in the first-line setting. Event-free survival was set as a dependent variable, and parameters for which *P* < 0.1 in the univariate analysis were independent variables. Forward selection based on a maximum likelihood ratio (Forward LR) was used as the regression method.

PBCT used as first-line chemotherapy (HR, 0.63, 95% CI 0.47 – 0.84; *P* = 0.002) together with ECOG score, number of metastatic organ sites, and time between breast surgery were observed to be independent predictive factors for PFS. We also found that cisplatin-based regimens were independent predictive factors for first-line PFS in the PBCT subgroup (HR, 0.59, 95% CI 0.35 – 0.99; *P* = 0.046) (Table 3).

**Table 3:** Factors independently predicting PFS and OS

*Cox regression results of PFS (n = 364)*			
Independent predictive factors	Hazard ratio (HR)	95% CI	*P* value
ECOG			
0	Ref		
1–2	1.53	1.12 – 2.09	0.008
Number of metastatic organ sites			
1	Ref		
2	1.21	0.89 – 1.65	0.224
≥3	1.78	1.30 – 2.44	<0.001
Time between breast surgery and recurrent disease			
<12 months	Ref		
≥12 months	0.58	0.44 – 0.75	<0.001
PBCT used as first-line chemotherapy			
No	Ref		
Yes	0.63	0.47 – 0.84	0.002
***Cox regression results of PFS in subgroup of PBCT as the first line chemotherapy (n = 218)***			
ECOG			
0	Ref		
1–2	1.52	1.06 – 2.18	0.022
Number of metastatic organ sites			
1	Ref		
2	1.47	1.01 – 2.13	0.045
≥3	2.23	1.53 – 3.25	<0.001
Time between breast surgery and recurrent disease			
<12 months	Ref		
≥12 months	0.63	0.46 – 0.86	0.004
Type of PBCT			
Non-cisplatin-based	Ref		
Cisplatin-based	0.59	0.35 – 0.99	0.046
***Cox regression results of OS (n = 379)***			
Number of metastatic organ sites			
1	Ref		
2	1.32	0.94 – 1.86	0.108
≥ 3	2.67	1.92 – 3.71	<0.001
Time between breast surgery and recurrent disease			
< 12 months	Ref		
≥ 12 months	0.43	0.32 – 0.57	<0.001
Total lines of PBCT received			
< 2	Ref		
≥ 2	0.73	0.56 – 0.94	0.016

Abbreviations: PBCT, platinum-based chemotherapy; PFS: progression-free survival; OS: overall survival; CI: confidence interval; Ref: reference category.

Regarding OS, lines of PBCT (<2 line vs. ≥2 lines, HR, 0.73, 95% CI 0.56 – 0.94; *P* = 0.016) together with number of metastatic organ sites, and time between breast surgery were independent predictive factors for OS (Table 3).

## DISCUSSION

The controversy over the role of platinums in mTNBC persists, so we retrospectively re-evaluated this concept in a patient population, focusing on first-line and later line therapy. We found that PBCT, especially a cisplatin-based regimen, was significantly more effective than non-PBCT strategies in terms of PFS and ORR during first-line therapy in a metastatic setting, but OS did not improve. Also, no significant difference was observed in OS whether PBCT was introduced or not during treatment. Early and late introduction of PBCT did not influence OS, but more treatment lines (≥2) of PBCT significantly lengthened OS.

Both BRCA-associated breast cancer and sporadic triple-negative or basal-like breast cancers have characteristics consistent with abnormal DNA repair and genome-wide instability [23, 24], which lends support to the use of DNA-damaging compounds such as platinums. That PBCT yielded higher response and longer PFS than non-PBCT in our study established this concept and was in accordance with data from the phase II study by Fan's group [17] and the prospective phase III CBCSG006 [18]. Similar to our study, these two trials were based on Asian population. Differences in data from the TNT trial [19] which compared carboplatin with docetaxel and mainly included western populations suggest different types of platinums and different patient ethnicities may contribute to these differences. Not including the phase II study by Fan [17] which studied a relatively small sample size, OS benefits were not gained with first-line PBCT in our study and the TNT trial. For CBCSG006, although OS data are immature, death events were almost equal between arms. We do agree that post-progression crossover to the PBCT might contribute to no differences observed in OS because our study indicated that OS was not significantly influenced by the timing (early or late) of introduction of platinum for patients with only one line PBCT during the whole treatment. However, OS did not differ between groups with PBCT introduced or not during treatment, suggesting that re-evaluating the role of platinums in mTNBC is needed. For mTNBC patients with more extensive, rapidly progressive, or symptomatic disease, PBCT was preferred because—even without improved OS—PBCT appeared to offer more symptom control and allowed later line treatments. Identification of predictive markers for PBCT and population enrichment with those who benefit from PBCT should prolong OS for mTNBC patients.

Cisplatin-based regimens appeared to be significantly more efficacious than other platinum types as a first-line therapy in our study. Both PFS (8.0 months vs. 4.3 months) and ORR (59.6% vs. 35%) were comparable to data from the two phase III trials (TNT and CBCSG006) and other previous studies, findings which support our data. Compared with carboplatin, cisplatin has superior efficacy as a neo-adjuvant for locally advanced TNBC, with more patients achieving a pathological complete response and significant improvement in OS [22]. Also in the metastatic setting, more patients respond to cisplatin than carboplatin [21].

An important finding in our study was that more lines of PBCT improved OS and reduced the risk of death by 27% according to a Cox hazards model. Patients who received ≥2 lines of PBCT had a median OS more than 2 years (25.1 months), which suggested that the cumulative effect of better PFS from different lines of PBCT might ultimately result in longer OS.

Multivariate analysis confirmed that PBCT used as first-line chemotherapy was an independent predictive factor for PFS. For patients with first-line PBCT, a cisplatin-based regimen was a favorable predictive factor for longer first-line PFS. These data confirmed a potential role for platinums to treat mTNBC but they have not been confirmed to predict better OS. Through molecular profiling, TNBC was identified to be a disease of many intrinsic molecular subtypes (basal-like, claudin-low or human epidermal growth factor receptor 2 [HER2]-enriched) [25]. The high heterogeneity of TNBC suggests that platinums used to treat unselected mTNBC patients to improve OS may not be a good strategy. Application of the PAM50 intrinsic subtype signature to sub-classify TNBC groups as basal- and non-basal-like, or a determination of the status of BRCA1, BRCA2 and BRCAness may be better for testing platinums but this requires future study.

Our study is limited by its retrospective nature and potential patient and treatment selection biases, its inclusion of one institution and the fact that different baseline information as well as various intrinsic molecular subtypes of TNBC might lead to clinical heterogeneity. In addition, the impossibility to retrieve enough information to analyze the safety profile and tolerability of these PBCT regimens should be considered, with the solely exception of a fraction of patients treated in the most recent years, owing to the wide time window considered. Based on currently available, albeit incomplete, data we did not observe any unexpected warnings in terms of toxicity and adherence to these PBCT therapies. Even so, our study is the first and the largest re-evaluation of the role of PBCT in first-line and later line settings.

In conclusion, compared with non-PBCT, PBCT especially cisplatin-based regimens using at the first line can significantly improve ORR and PFS, but cannot improve OS. PBCT with only 1 line during the whole treatment might not be necessary for unselected mTNBC with the exception of an urgent demand to control disease or symptoms, however, ≥2 lines of PBCT did prolong OS. A better future strategy may be identification of predictive markers of PBCT for mTNBC patients.

## PATIENTS AND METHODS

### Patient selection

Retrospective analysis was conducted within a cohort of mTNBC patients who received PBCT or non-PBCT at FUSCC between July 2000 and March 2014. Data were collected from electronic patient records and hospital charts. Study procedures were approved by institutional ethical board of FUSCC. The eligible patients were ≥ 18 years old and had TNBC histologically confirmed at the primary tumor, with clinical, imaging, histological or cytological evidence of metastatic disease. Patients were classified as TNBC based on their surgical or biopsy results, histologically confirmed estrogen receptor (ER)-, progesterone receptor (PR)-, and HER2- were defined as follows: ER- and PR- were defined as < 1% positive tumor cells immunohistochemical nuclear staining and HER2- was defined as having an IHC score of 0 or 1+ or a FISH non-amplified score according to ASCO guidelines. Patients with incomplete receptor status or changed status in metastatic lesions inconsistent with the definition described above were excluded.

### Data collection

The efficacy of PBCT and non-PBCT was analyzed according to ORR, PFS, and OS. Tumor response was evaluated in accordance with the Response Evaluation Criteria in Solid Tumors (RECIST 1.1) guidelines by computed tomography scanning or magnetic resonance imaging if indicated. The date of disease progression was determined from the clinical notes. PFS was defined as the time from the start of the treatment until disease progression or death. OS was calculated from the start of the first-line treatment to death by any cause or censored at the last date the patient was known to be alive.

One of the objectives of this study was to establish prognostic factors for outcomes in TNBC patients who received PBCT. Data for clinical variables identified through literature review were collected as potential important predictors of ORR, PFS and OS in mTNBC patients. Potential prognostic variables collected at the time of diagnosis of distant metastases or the beginning of treatment included age, menstruation status, details about neo-adjuvant and adjuvant chemotherapy administered, distant disease-free interval, and number and sites of metastatic disease.

### Statistical analysis

Results were analyzed using SPSS 16.0 (SPSS, Chicago, IL). Binary logistic regression model was used to identify the factors that independently influence first-line ORR. Cox proportional hazards models were used to estimate adjusted hazard ratios (HRs) and 95% CIs of disease progression or death. The models were adjusted for age, menstruation status, prior adjuvant and neo-adjuvant chemotherapy, site and extent of first distant relapse, and presence of visceral metastasis. Median PFS and OS were all estimated by the Kaplan–Meier method and compared using a log-rank test. Response rates of different regimens were compared using a chi-squared test or Fisher's exact test. All *P* values were two sided, *P* < 0.05 was considered to indicate a statistically significant difference.

## Abbreviations

TNBC: triple-negative breast cancer
ER: estrogen receptor
PR: progesterone receptor
HER2: human epidermal growth factor receptor 2
DFI: disease-free interval
BLBC: basal-like breast cancer
PBCT: platinum-based chemotherapy
ORR: overall response rate
PFS: progression-free survival
OS: overall survival
FUSCC: Fudan University Shanghai Cancer Center
RECIST: Response Evaluation Criteria in Solid Tumors
SPSS: Statistical Product and Service Solutions.
